# Enhancing the Production of the Fungal Pigment Aurofusarin in *Fusarium graminearum*

**DOI:** 10.3390/toxins10110485

**Published:** 2018-11-21

**Authors:** Klaus Ringsborg Westphal, Rasmus Dam Wollenberg, Florian-Alexander Herbst, Jens Laurids Sørensen, Teis Esben Sondergaard, Reinhard Wimmer

**Affiliations:** 1 Department of Chemistry and Bioscience, Aalborg University, 9220 Aalborg, Denmark; kw@bio.aau.dk (K.R.W.); rwo@bio.aau.dk (R.D.W.); fah@bio.aau.dk (F.-A.H.); tes@bio.aau.dk (T.E.S.); 2 Department of Chemistry and Bioscience, Aalborg University, 6700 Esbjerg, Denmark; jls@bio.aau.dk

**Keywords:** aurofusarin, PKS12, secondary metabolites, polyketide, natural products, *aurR1*, *Fusarium*, pigment, fungi, mycotoxins

## Abstract

There is an increasing demand for products from natural sources, which includes a growing market for naturally-produced colorants. Filamentous fungi produce a vast number of chemically diverse pigments and are therefore explored as an easily accessible source. In this study we examine the positive regulatory effect of the transcription factor AurR1 on the aurofusarin gene cluster in *Fusarium graminearum*. Proteomic analyses showed that overexpression of AurR1 resulted in a significant increase of five of the eleven proteins belonging to the aurofusarin biosynthetic pathway. Further, the production of aurofusarin was increased more than threefold in the overexpression mutant compared to the wild type, reaching levels of 270 mg/L. In addition to biosynthesis of aurofusarin, several yet undescribed putative naphthoquinone/anthraquinone analogue compounds were observed in the overexpression mutant. Our results suggest that it is possible to enhance the aurofusarin production through genetic engineering.

## 1. Introduction

The fungal polyketides are a diverse group of natural products that comprises of mycotoxins, clinical important drugs, and pigments [1,2,3]. Examples of the first two are fusarielins and lovastatin, which are shown to stimulate MCF-7 cell proliferation or commercially used against hypercholesterolemia, respectively [4,5,6]. The pigment aurofusarin, which is the target of this study, is a homodimeric naphthoquinone produced by crop molds of the genus *Fusarium* and is often associated with naturally infected wheat where the compound can occur in high levels [7].

Aurofusarin was initially described in 1937 as a golden yellow micro-crystalline pigment but the chemical structure was not elucidated until 1966 [8,9,10,11]. The *PKS12* gene cluster from *Fusarium graminearum* was identified to be responsible for aurofusarin biosynthesis under the control of the local transcription factor AurR1 [12,13]. Kim and colleagues showed that the knock-out of *aurR1* resulted in complete absence of pigmentation and that re-integration of a constitutively expressed *aurR1* gene into the knock-out mutant led to increased aurofusarin production compared to the wild-type (wt) [12]. 

Besides *aurR1*, the *PKS*12 gene cluster contains ten genes encoding a polyketide synthase (PKS12), a rubrofusarin pump (AurT), a laccase not required for aurofusarin production (AurL2), a second transcription factor (AurR2), and six tailoring enzymes [14]. The transcription factor AurR2 possibly acts as a co-regulator of the *PKS12* gene cluster. Deletion of *aurR2* results in an altered ratio of rubrofusarin to aurofusarin [14].

The *PKS12* gene cluster is responsible for the production of several other compounds including YWA1, rubrofusarin, and fuscofusarin [15,16]. The aurofusarin biosynthetic pathway can be redirected to produce citreoisocoumarin by changing nitrogen sources and pH [17]. Further, the laccase catalyzing monomeric activation resulting in radical-mediated dimerization could potentially lead to several other dimerization patterns than that present in aurofusarin [14].

Naphthoquinones, including dimeric naphthoquinones, have previously been described as having antibiotic activity against both Gram-positive and Gram-negative bacteria, as well as yeast of the genus *Candida* [18,19]. In fact, 22 naphthoquinones from *F. solani* and *F. oxysporum* were examined by Baker and colleagues, of which 12 were antimicrobial against *Streptococcus pyogenes* and 15 were active against *Staphylococcus aureus* [20]. Aurofusarin was shown to inhibit probiotic bacteria, which might shift the gut microbes to a more harmful constitution causing illness and diarrhea [21]. Also, aurofusarin is cytotoxic, genotoxic, and induces oxidative stress in human colon cells [22].

Cytotoxicity and antibiotic activity of naphthoquinones from the *PKS12* pathway of *F. graminearum* should be more thoroughly investigated, including animal trials to assess aurofusarin’s antibacterial/toxicological effect when ingested through feed. However, this requires large quantities of the compound, which is very expensive. To accommodate this, we aimed to make aurofusarin and derivatives thereof more readily available. The wt *F. graminearum* produces aurofusarin under several growth conditions, often as one of the most abundant secondary metabolites. Therefore, we chose an over-expression strategy that contributed to the natural expression machinery by additionally expressing the *aurR1* gene in an ectopic position close to the β-tubulin locus. Furthermore, we describe a method for pigment extraction directly from liquid growth media, resulting in the detection of four putative yet undescribed naphthoquinones.

## 2. Results and Discussion

**Figure 1:**
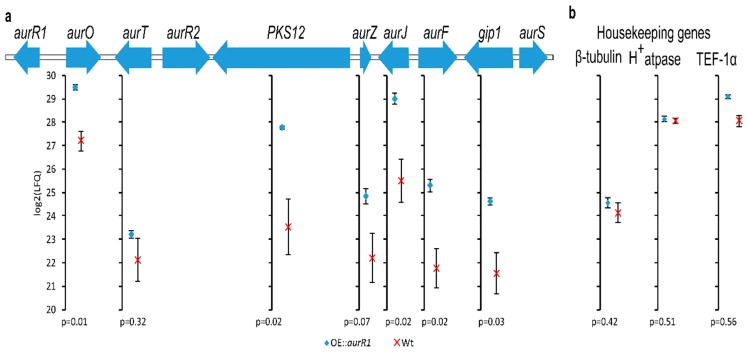
The *PKS12* gene cluster (**a**) and housekeeping genes (**b**) with mean log2 (label-free relative quantification) intensities of identified proteins and standard error bars calculated from three biological replicates of OE::*aurR1* and wt grown on Cz medium. The *p*-values are calculated from a two-tailed Student’s *t*-test. Missing/undetected values from the wild type were imputed to reflect low-abundant proteins.

AurR1 is a positive-acting transcription factor, influencing the expression of several genes in the aurofusarin gene cluster. To investigate the upregulation of the *PKS12* gene cluster, the transcription factor *aurR1* was expressed in an ectopic position next to the β-tubulin gene. The transcription factor was under control of the constitutive TEF promotor. The resulting OE::*aurR1* mutant was sequenced to approximately 200× coverage (Supplementary Figure S1) to verify the correct integration of the insert. To determine the protein expression level, protein was extracted from dried mycelial powder of both OE::*aurR1* and the wt and analyzed using shotgun proteomics. Seven out of ten proteins from the aurofusarin gene cluster were detected, of which five showed significantly increased expression (*p* < 0.05) in the OE::*aurR1* mutant when comparing label-free quantification (LFQ) values (Table 1 and Figure 1a) [23]. We also compared three housekeeping genes to evaluate the performance of the method for which we observed no difference between the mutant and the wt. These were β-tubulin (UniProt ID: I1RR95), H^+^-ATPase (UniProt ID: I1RCU4), and TEF-1α (UniProt ID: I1R.W.X5) with *p*-values calculated to be 0.4, 0.5, and 0.5, respectively (Figure 1b). The *p*-value for AurZ was calculated to be 0.07 and was therefore slightly above the threshold for statistical significance. However, two out of three LFQ values for AurZ in the wt were imputed to reflect low abundant proteins, which might have caused an overestimation of the actual *p*-value [24]. There was no statistical significant difference in expression for AurT (*p* = 0.32) between the mutant and the wt, indicating either that AurR1 does not affect the transcription of *aurT* or, perhaps more likely, the applied method for protein extraction does not favor membrane bound proteins [25]. The mutant to the wt LFQ ratios for detected proteins from the cluster were calculated to be between 4.5 and 11.2, with the exception of AurT, which was at 1.6. Expectedly, AurR1 and AurR2 were not detected. Transcription factors are known to have unstable mRNA and proteins, both contributing to low abundance [26]. Additionally, detection of compartmentalized and DNA binding proteins, such as transcription factors, benefit from selective purification and enrichment procedures [27]. AurL2 and AurS were not detected, probably due to low abundance. It could be expected that the expression of *aurL2* would not be influenced by AurR1 as it is not required for aurofusarin biosynthesis [14]. AurS contains a transmembrane helix (InterPro [28] and Pfam [29]) indicating that this protein is anchored to the cell membrane, which could lead to reduced solubility and yield in the protein extraction.

**Figure 2:**
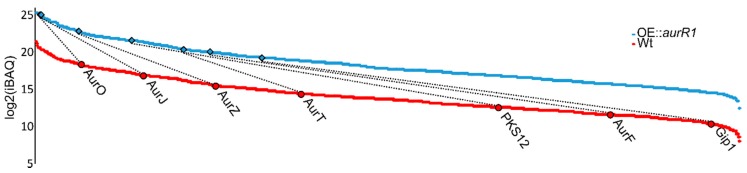
Scatter plots of 908 identified proteins sorted from highest to lowest log2 (intensity-based absolute quantification) intensities calculated from three biological replicates of OE::*aurR1* and the wt grown on a Cz medium. The proteins from the PKS12 gene cluster are highlighted and the dotted lines show the correlation between OE::*aurR1* and the wt. The wt plot is downshifted by five log2 units.

When comparing intensity-based absolute quantification (iBAQ) values, the most abundant protein from the *PKS12* cluster in both the wt and the mutant was AurO closely followed by AurJ (Figure 2) [26]. In the mutant, these two proteins are expressed as the sixth and eighth most abundant within the 908 proteins quantified. Comparing OE::*aurR1* and the wt, all observed proteins of the *PKS12* cluster have become more abundant compared to other cellular proteins. If one sorts the proteins or the *PKS12* cluster by abundance, their relative order is maintained when going from the wt to OE::*aurR1*, with the exception of AurF which went from sixth in the wt to third in the mutant. As the *p*-values in Table 1 already indicate, there is no significant difference in expression for AurT between the mutant and the wt. Either AurR1 does not affect the expression of *aurT*, or AurR1 affects the expression of *aurT*, albeit to a lesser extent compared to the other proteins in the cluster.

**Figure 3:**
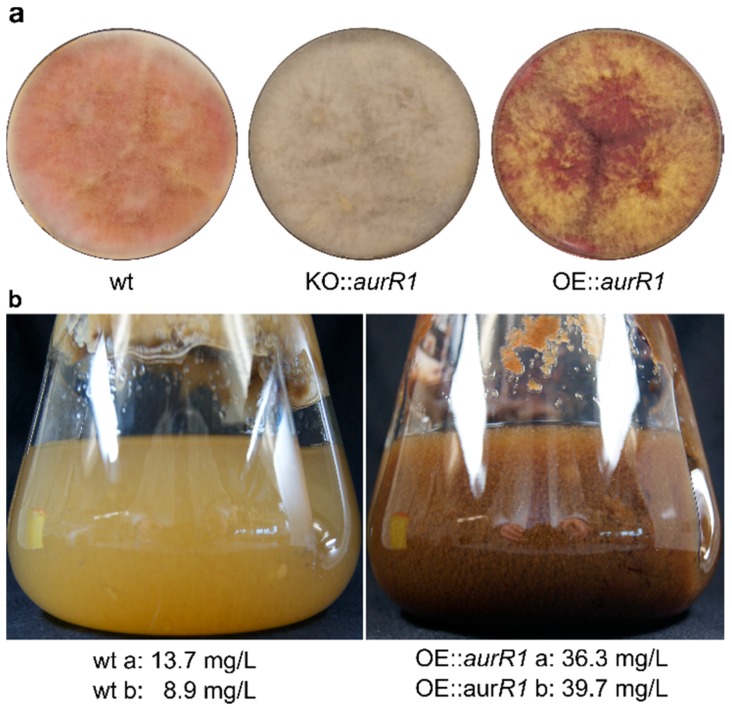
Color differences of wild type and mutants. (**a**) Wild type *Fusarium graminearum* (wt), *aurR1* knockout mutant (KO::*aurR1*), and OE::*aurR1* grown on a Cz agar medium for 6 days. (**b**) Cultures of wt and OE::*aurR1* grown for 3 days in a liquid Cz medium. ^1^H-NMR quantification of aurofusarin from biological duplicates are indicated in mg/mL.

The phenotype of the OE::*aurR1* mutant appeared deep red with yellow aerial mycelium when grown for six days on a solid Cz medium (Figure 3a) compared to the wt, which was pale red/pink with white aerial mycelium. An *aurR1* knockout mutant previously described by Frandsen and colleagues in 2006 was cultured simultaneously and, as expected, exhibited complete lack of coloration [14]. Cultures of OE::*aurR1* and the wt were grown in liquid Cz medium for three days, at which point a clear difference in coloration was observed (Figure 3b). The wt culture was pale-yellowish while the mutant displayed a dark red-brownish color. Aurofusarin, and derivatives thereof, contain conjugated systems absorbing visible light at longer wavelengths, which could explain the observed difference. Metabolites were extracted directly from the medium from two individual 1 L cultures of both the mutant and the wt grown for three days and the concentration of aurofusarin was measured using quantitative ^1^H-NMR. Expectedly, the mutant produced the most aurofusarin with concentrations calculated at 36.3 and 39.7 mg/L compared to 8.9 and 13.7 mg/L for the wt. The mutant thereby produced between 2.6 and 3.4 times more aurofusarin than the wt, supporting the proteomics data.

The final step in the aurofusarin biosynthetic pathway is a dimerization of rubrofusarin to aurofusarin, which is predicted to be catalyzed by an extracellular enzyme complex consisting of Gip1, AurF, AurO, and AurS [15]. Gip1 is a predicted laccase with a multicopper oxidase domain and we therefore hypothesized that the availability of copper ions could influence aurofusarin production in the overexpression mutant. However, ranging the concentration from 10 to 80 µM CuSO_4_ × 5H_2_O did not significantly affect aurofusarin production. The highest average production was observed at 10 µM CuSO_4_ × 5H_2_O, which yielded 270 mg/L aurofusarin after seven days of growth (Supplementary Figure S2).

**Figure 4:**
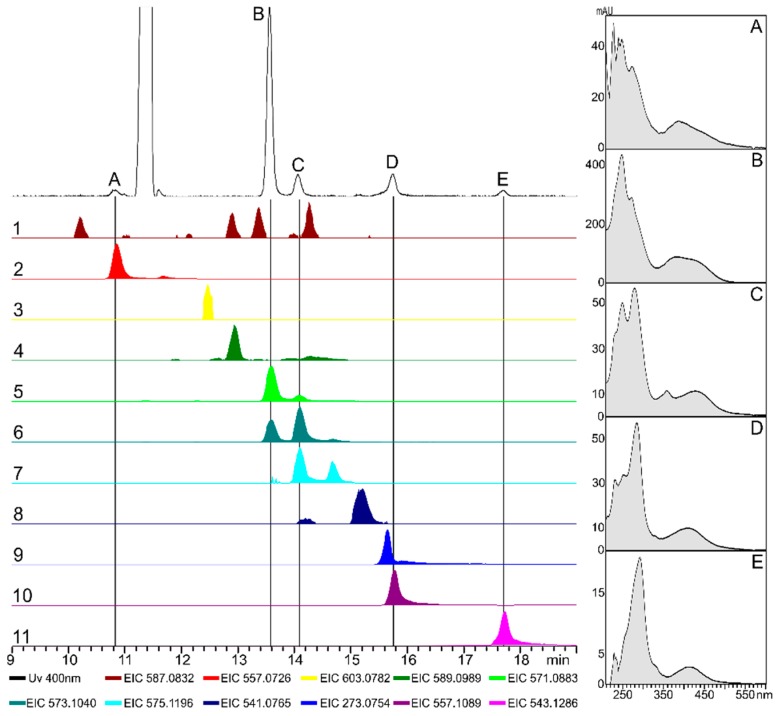
Chromatograms from a metabolite extract of Cz medium 5 days after inoculation with OE::*aurR1*. Left: a UV 400 nm chromatogram (spectral background subtracted) with selected peaks (A–E) and underlying colored extracted ion chromatograms (EICs) of selected masses (1–11, [M + H]^+^ ± 0.02 Da) scaled by relative intensities. Right: UV spectra (205–600 nm) for peaks A–E.

The extracted compounds from the OE::*aurR1* culture were analyzed using HPLC-DAD-HRMS (Figure 4). Five peaks (A-E) were observed from a UV chromatogram (400 nm) with UV spectra similar to that of aurofusarin (spectrum B). The UV spectrum of aurofusarin has two peak maxima at 244 and 268 nm and a broad peak at 381 nm, with a UV(244)/UV(268) absorption ratio around 1.5. A similar ratio was observed for peak A; however, the ratio had shifted to 0.9 for peak C. The absorption ratios for peak D and E were calculated at 0.6 and 0.4, respectively. Eleven masses were observed, which corresponded to compounds with chemical formulae comparable to that of aurofusarin or possible analogues (Table 2). Peak B had a [M + H]^+^ at 571.09 Da corresponding to aurofusarin. Further, the [M + H]^+^ for peak D was measured at 557.11 Da corresponding to fuscofusarin. Peak A aligned with a [M + H]^+^ of 557.07 Da fitting the chemical formula C_29_H_16_O_12_, which is comparable to that of aurofusarin with the loss of CH_2_. This could be explained by one of the monomers of aurofusarin lacking an O-methylation, which could also explain the lower retention time. Peak C was measured at 573.10 Da [M + H]^+^ corresponding to aurofusarin with an increased mass of two hydrogens. Finally, peak E had a mass at 543.13 giving a chemical formula of C_30_H_22_O_10_. Compared to aurofusarin, this compound has an increase of four hydrogen atoms and a loss of two oxygen atoms. This could be explained by a homodimerization of rubrofusarin, which was also proposed by Frandsen in 2006 [14]. The remaining six masses [M + H]^+^ were measured at 587.08 Da (C_30_H_18_O_13_, aurofusarin + O), 603.08 Da (C_30_H_18_O_14_, aurofusarin + 2O), 589.10 Da (C_30_H_20_O_13_, aurofusarin + H_2_O), 575.12 Da (C_30_H_22_O_12_, aurofusarin + 4H), 541.08 (C_29_H_16_O_11_, aurofusarin − CH_2_O), and 273.08 Da (rubrofusarin). When over-expressing a local transcription factor from a secondary metabolite cluster, it could be expected that the natural ratio and abundance of modifying enzymes was changed, which might result in alternative routes for the biosynthetic machinery. This could explain the proposed naphthoquinone derivatives observed from the OE::*aurR1* metabolite extract. A database search for known naphthoquinone/anthraquinone natural products employing Reaxys and PubChem could not identify any possible candidates for peaks 1–4 indicating that these might be yet undescribed compounds.

## 3. Conclusions

In the present study, we have described the changes of protein expression and secondary metabolite production when the local transcription factor AurR1 is over-produced. Five of the seven detected proteins from the *PKS12* cluster were observed to be significantly increased in the AurR1 over-producing mutant. In general, all proteins from the cluster were more abundant relative to the total cell protein in the mutant compared to the wt; however, the transporter AurT was affected to a lesser extent. The mutant produced between 2.6 and 3.4 times the amount of aurofusarin compared to the wild type with a maximal observed mean production of 270 mg/L. The availability of copper ions did not affect aurofusarin production, but future studies with other factors, such as a nitrogen and carbon source, temperature, and aeration can perhaps be more successful. The mutant was shown to produce several compounds with chemical formulae comparable to aurofusarin analogues, four of which had no match to known naphthoquinone/anthraquinone natural products. The extraction of aurofusarin and related compounds directly from the media enabled faster and more efficient production of aurofusarin, which could easily be implemented to flow reactors.

## 4. Materials and Methods

### 4.1. Generation and Verification of aurR1 Overexpression Mutant

*aurR1* (FGSG_02320) was amplified using PCR using the primers PaurR1_Fw (5′-ACGTCGGATCCATGAGTTCCACAGACCCCCTTCT-3′) and PaurR1_Rv (5′-ACGTCTCTAGACTACTTGACCTCGAGCTTCTTTCGT-3′) (Eurofins Genomics, Ebersberg, Germany) holding restriction sites for BamHI and XbaI, respectively. PCR was conducted with PfuTurbo Cx Hotstart DNA Polymerase (Agilent, Santa Clara, CA, USA) using genomic DNA from *F. graminearum* PH-1 (NRRL 31084) as a template. The amplified *aurR1* PCR fragment was cloned into a U-GOTL vector, which is a modified version of the U-GOAL vector [30]. The U-GOTL vector has border regions targeting the β-tubulin locus. The cloned vector was verified using colony PCR and sequencing of the integrated site by Eurofins Genomics.

The vector was electroporated into *Agrobacterium tumefaciens* LBA4404 obtained from Invitrogen, Thermo Fisher Scientific Inc. catalog number: 18313-015, which was subsequently used to transform macroconidia of *F. graminearum* as previously described [31]. The macroconidia were obtained from a 5-day culture grown in a carboxymethylcellulose medium (CMC) (15 g/L sodium carboxymethylcellulose medium viscosity (MP Biomedicals, Solon, OH, USA), 1 g/L NH_4_NO_3_, 1 g/L KH_2_PO_4_, 0.5 g/L MgSO_4_·7H_2_O, 1 g/L yeast extract (Difco Lboratories, Detroit, MI, USA)) in the dark at 20 °C with 150 rpm. The *aurR1* overexpression mutants were selected for by growing on a defined *Fusarium* medium containing 300 µg/mL of geneticin (Sigma-Aldritch, St. Louis, MO, USA), and the correct insertion of the *aurR1* gene was verified by full genome sequencing.

Fungal DNA was extracted with the FastDNA SPIN Kit for Soil (MP Biomedicals) and cleaned with Agencourt AMPure XP beads (Beckman Coulter, Brea, CA, USA).

A Nextera paired-end DNA library (Illumina, San Diego, CA, USA) was prepared according to the manufacturers description, with the modification of using Agencourt AMPure XP beads (0.7 bead:sample ratio) for the post-tagmentation clean-up. The quality of the purified DNA was measured using a NanoDrop ND-1000 (Thermo Scientific, Waltham, MA, USA), a D1000 ScreenTape system (Agilent, Santa Clara, CA, USA), and using the Qubit dsDNA HS assay kit (Thermo Scientific, USA).

The paired-end library was sequenced to approximately 200× coverage on the Illumina HiSeq 2500 system using the Rapid SBS Kit v2 (2 × 250 cycles). The data were trimmed for low quality reads, ambiguous nucleotides, and adaptors in CLC Genomics Workbench v. 9.4.2 (Qiagen, Vedbæk, Denmark). Following read-merging, reads were mapped to a reference genome (PH1) and further assessed for random insertion using a de novo assembly approach.

### 4.2. Proteomics

Triplicate cultures of OE::*aurR1* and the wild type were grown in Liquid Czapek Dox (Cz) medium (30 g/L sucrose, 3 g/L NaNO_3_, 1 g/L K_2_HPO_4_, 0.5 g/L MgSO_4_·7H_2_O, 0.5 g/L KCl, 0.01 g/L FeSO_4_·7H_2_O, 16 mg/L ZnSO_4_·7H_2_O, 5 mg/L CuSO_4_·5H_2_O, pH 6.25) at 25 °C with 100 rpm for five days. The cultures were filtered through MiraCloth (Calbiochem, Merck, Darmstadt, Germany) and the fungal mycelium was lyophilized. Protein concentration was determined from 10 mg of dried fungal powder suspended in 500 µL of cold sterile H_2_O. The solution was heated to 95 °C for 10 min and afterwards cooled on ice. The samples were centrifuged for 5 min at 14,100× *g*, and the supernatant was transferred to new tubes. The samples were centrifuged a second time for 5 min at 14,100× *g* and the protein concentration was measured using Qubit. 

Samples for the proteomic analyses were processed using a polyvinylidene fluoride membrane-based proteomic sample preparation approach as described elsewhere [32]. In short, approximately 20 µg of protein was suspended in urea buffer for denaturation, and was reduced and carbamidomethylated before in-solution trypsin digestion. Clean-up was performed by washing and elution from the membrane. Tryptic peptides were measured using nLC-MS/MS (Ultimate 3000 coupled to a Q Exactive, Thermo Fisher Scientific, Waltham, MA, USA) applying a 3 h method (≈140 min elution window), where details can be found elsewhere [33]. Mass spectra were analyzed using MaxQuant (version 1.5.3.30, Max Planck Institute of Biochemistry, Martinsried, Germany) [34] as previously described [33]. Besides activation of label-free quantification by the MaxQuant label-free quantification algorithm [35] and iBAQ calculations [26], settings were kept on default. This includes a false-discovery rate of maximum 1% on peptide and protein level. An organism-specific protein database in FASTA file format was obtained from UniProt [36].

The LFQ and iBAQ data matrix’s were imported into Perseus (version 1.5.6.0, Max Planck Institute of Biochemistry, Martinsried, Germany) [37] and grouped into either OE::*aurR1* or wt. The data was filtered to remove proteins that were only identified by site, reverse, and potential contaminants. Further, all proteins must be represented by at least two valid values larger than 0 in at least one of the groups. The values were log2(x) transformed, and missing values were imputed from a normal distribution of the whole matrix using a width of 0.3 and a downshift of 1.8 to represent low abundant proteins. A two-tailed Student’s *t*-test was applied to determine whether protein concentration levels differed significantly between the OE::*aurR1* and wild type cultures. Statistical significance was defined as a *p*-value ≤ 0.05.

### 4.3. Metabolite Extraction from Liquid Growth Medium, NMR Quantification, and LCMS

One L Cz media was inoculated with 5 × 10^3^ spores of OE::*aurR1* or the wild type and incubated in a 2 L Erlenmeyer flask with three baffles at 25 °C and 100 rpm for 3 days in complete darkness, both in duplicates. The growth media was filtrated through Miracloth. 300 mL media in a 1 L bluecap bottle, and was acidified with 20 mL 5 M HCl and shaken. The color of the media was observed to change from dark brown to yellow. Three hundred milliliters of chloroform was added and the mixture was shaken. After settling, the color could be observed in the water phase. Two hundred milliliters of methanol was added and the mixture was shaken vigorously. The phases were allowed to settle and a yellow coloration was now observed in the organic phase. Most of the water phase was carefully decanted. Two hundred milliliters of 10% *w*/*v* NaCl in demineralized water was added to the chloroform together with 100 mL of methanol. The mixture was shaken and the phases were allowed to settle. The organic phase was collected using a separating funnel. The solvent was removed using rotary evaporation in vacuo at 40 °C.

The dried metabolite extracts were re-dissolved in 12 mL of chloroform and analyzed using LC-MS. Sixty microliters of each sample were dried under N_2_ gas and dissolved in 600 µL chloroform-d (99.8 atom % D, 0.03% TMS, Euriso-Top GmbH, Saarbrücken, Germany) and analysed by NMR. 

^1^H and ^1^H-PULCON [38] spectra were recorded on a Bruker AVIII-600 MHz spectrometer (Bruker, Karlsruhe, Germany) equipped with a triple resonance cryogenically cooled probe with z-gradients and controlled by TopSpin 3.5pl6. All recordings were performed in chloroform-d at 298.1 K and calibrated to TMS ^1^H (0 ppm). 

Extracts from OE::*aurR1* were analyzed using HPLC-DAD-HRMS on a Hitachi Elite LaChrom HPLC system (Hitachi Ltd., Tokyo, Japan) equipped with an L-2130 pump, L-2200 autosampler, L-2300 column oven at 40 °C with a C6-phenyl column (150 × 4.6 mm Ascentis Xpress 2.7 μm, Sigma-Aldrich, St. Louis, MO, USA), and an L-2450 DAD detector. The system was coupled through a 5:95 flowsplitter to a high-resolution mass spectrometer (Compact qTOF, Bruker, Karlsruhe, Germany) with an electrospray source (Capilary: 4500 V; end plate offset 500 V; dry gas 4.0 L/min, 200 °C) and run in positive mode. The 1 mL/min gradient initiated at 90% solvent A (water, HiPerSolv, VWR, Radnor, PA, USA) and 10% solvent B (acetonitrile, Hypersolve, VWR, Radnor, PA, USA), were both supplemented with 0.1% formic acid (MS grade 98%, Sigma-Aldrich), increasing linearly to 100% solvent B over 20 min and held for 5 min. Twelve seconds after sample injection, a 2 µL calibrant solution (10 mM NaOH and 26 mM formic acid in 1:1 MS-grade H_2_O:isopropanol) was injected into the mass spectrometer.

### 4.4. Production of Aurofusarin in Response to Copper Availability

Prior to experiments, spores of OE::*aurR1* were produced in 50 mL of liquid CMC medium by shaking at 150 rpm and 20 °C for four days [39]. The macroconidia were resuspended in sterile H_2_O after two rounds of centrifugation to reach a concentration of 3.5 × 10^6^/mL. One hundred microliters of the spore solution was added to 250 mL baffled flasks containing 100 mL of Czapek dox medium [40] to which 0.1 mL ZnSO_4_ × 7 H_2_O (1 g/100 mL) was added together with 0.1 mL CuSO_4_ × 5 H_2_O (0.25, 0.5, 1, 2, 3, or 4 g/100 mL). The flasks were incubated in a dark shaking incubator at 120 rpm for one week at 25 °C.

To determine the aurofusarin production, 1 mL of medium was transferred to a micro centrifuge tube and spun for two minutes at 12,000 rpm to remove impurities. A 10 µL sample was then transferred to an HPLC vial and diluted with 990 µL of methanol. Aurofusarin was quantified using HPLC-MS/MS as given previously using a two-fold dilution series ranging from 0.019–10 mg/L [41,42].

## Figures and Tables

**Table 1:** The PKS12 gene cluster in *F. graminearum*. Predicted protein function by Pfam or InterPro.

Locus	Name	UniProt ID	Predicted Function	Mutant (LFQ)/Wt (LFQ)
FG02320	AurR1	I1RF54	Positive acting transcription factor	
FG02321	AurO	I1RF55	Oxidoreductase	4.5
FG02322	AurT	I1RF56	Rubrufusarin pump	1.6
FG02323	AurR2	I1RF57	Transcription factor	
FG02324	PKS12	I1RF58	Polyketide synthase producing YWA1	10.8
FG02325	AurZ	I1RF59	Dehydratase	4.6
FG02326	AurJ	I1RF60	*O*-methyltransferase	8.4
FG02327	AurF	I1RF61	Monooxygenase	11.2
FG02328	Gip1	I1RF62	Cu-oxidase	7.9
FG02329	AurS	I1RF63	Fasciclin-like domain containing protein	
FG02330	AurL2 ^1^	I1RF64	Cu-oxidase	

^1^ Is not required for aurofusarin production.

**Table 2:** List of selected masses from Figure 4 number 1–11 (#) including retention time (RT), observed charged mass ([M + H]^+^), proposed chemical formula, and the compound name/modification relative to aurofusarin.

#	RT (min)	Mass [M + H]^+^	Chemical Formula	Name/Variation
1	10.2; 12.9 ^1^; 13.4; 14.3	587.0820	^2^ C_30_H_18_O_13_	+O
2	10.9 (A); 11.7	557.0715	^2^ C_29_H_16_O_12_	−CH_2_
3	12.5	603.0769	^2^ C_30_H_18_O_14_	+2O
4	12.9	589.0977	^2^ C_30_H_20_O_13_	+H_2_O
5	13.6 (B); 14.1 (C)	571.0871	C_30_H_18_O_12_	aurofusarin
6	13.6 ^1^ (B); 14.1 (C)	573.1027	C_30_H_20_O_12_	+2H
7	14.1^1^ (C); 14.7	575.1184	C_30_H_22_O_12_	+4H
8	15.2	541.0765	C_29_H_16_O_11_	−CH_2_O
9	15.7	273.0758	C_15_H_12_O_5_	rubrofusarin
10	15.8 (D)	557.1078	C_30_H_20_O_11_	fuscofusarin
11	17.7 (E)	543.1286	C_30_H_22_O_10_	−2O + 4H

^1^ The observed EIC peak was caused by an isotopomer of a different compound or by a compound with a closely related mass. ^2^ Chemical formulae that does not match any natural products with a naphthoquinone/anthraquinone structure based on a Reaxys and PubChem database search.

## Supplementary Materials

The following are available online at http://www.mdpi.com/2072-6651/10/11/485/s1, Figure S1: Sequencing results for the OE::*aurR1* mutant, Figure S2: Production of aurofusarin in the OE::*aurR1* mutant in response to variable concentration of copper.

## References

[1] Sayyed I., Majumder D.R. (2015). Pigment Production from Fungi. Int. J. Curr. Microbiol. Appl. Sci..

[2] Peraica M., Radić B., Lucić A., Pavlović M. (1999). Toxic effects of mycotoxins in humans. Bull. World Health Organ..

[3] Aly A.H., Debbab A., Proksch P. (2011). Fifty years of drug discovery from fungi. Fungal Divers..

[4] Sondergaard T.E., Klitgaard L.G., Purup S., Kobayashi H., Giese H., Sørensen J.L. (2012). Estrogenic effects of fusarielins in human breast cancer cell lines. Toxicol. Lett..

[5] McKenney J.M. (1988). Lovastatin: A new cholesterol-lowering agent. Clin. Pharm..

[6] Henwood J.M., Heel R.C. (1988). Lovastatin. A preliminary review of its pharmacodynamic properties and therapeutic use in hyperlipidaemia. Drugs.

[7] Streit E., Naehrer K., Rodrigues I., Schatzmayr G. (2013). Mycotoxin occurrence in feed and feed raw materials worldwide: Long-term analysis with special focus on Europe and Asia. J. Sci. Food Agric..

[8] Ashley J.N., Hobbs B.C., Raistrick H. (1937). Studies in the biochemistry of micro-organisms: The crystalline colouring matters of *Fusarium culmorum* (W. G. Smith) Sacc. and related forms. Biochem. J..

[9] Shibata S., Morishita E., Takeda T., Sakata K. (1966). The structure of aurofusarin. Tetrahedron Lett..

[10] Baker P.M., Roberts J.C. (1966). Studies in mycological chemistry. Part XXI. The structure of aurofusarin, a metabolite of some Fusarium species. J. Chem. Soc. C.

[11] Shibata S., Morishita E., Takeda T., Sakata K. (1968). Metabolic Products of Fungi. XXVIII. The Structure of Aurofusarin. (2). Chem. Pharm. Bull..

[12] Kim J.E., Jin J., Kim H., Kim J.-C., Yun S., Lee Y. (2006). GIP2, a Putative Transcription Factor That Regulates the Aurofusarin Biosynthetic Gene Cluster in *Gibberella zeae*. Appl. Environ. Microbiol..

[13] Malz S., Grell M.N., Thrane C., Maier F.J., Rosager P., Felk A., Albertsen K.S., Salomon S., Bohn L., Schäfer W. (2005). Identification of a gene cluster responsible for the biosynthesis of aurofusarin in the *Fusarium graminearum* species complex. Fungal Genet. Biol..

[14] Frandsen R.J.N., Nielsen N.J., Maolanon N., Sørensen J.C., Olsson S., Nielsen J., Giese H. (2006). The biosynthetic pathway for aurofusarin in *Fusarium graminearum* reveals a close link between the naphthoquinones and naphthopyrones. Mol. Microbiol..

[15] Frandsen R.J.N., Schütt C., Lund B.W., Staerk D., Nielsen J., Olsson S., Giese H. (2011). Two novel classes of enzymes are required for the biosynthesis of aurofusarin in *Fusarium graminearum*. J. Biol. Chem..

[16] Klitgaard A., Frandsen R.J.N., Holm D.K., Knudsen P.B., Frisvad J.C., Nielsen K.F. (2015). Combining UHPLC-High Resolution MS and Feeding of Stable Isotope Labeled Polyketide Intermediates for Linking Precursors to End Products. J. Nat. Prod..

[17] Sørensen J.L., Nielsen K.F., Sondergaard T.E. (2012). Redirection of pigment biosynthesis to isocoumarins in Fusarium. Fungal Genet. Biol..

[18] Medentsev A.G., Akimenko V.K. (1998). Naphthoquinone metabolites of the fungi. Phytochemistry.

[19] Futuro D.O., Ferreira P.G., Nicoletti C.D., Borba-Santos L.P., Da Silva F.C., Rozental S., Ferreira V.F. (2018). The antifungal activity of naphthoquinones: An integrative review. An. Acad. Bras. Cienc..

[20] Baker R.A., Tatum J.H., Nemec S. (1990). Antimicrobial activity of naphthoquinones from Fusaria. Mycopathologia.

[21] Sondergaard T.E., Fredborg M., Oppenhagen Christensen A.M., Damsgaard S.K., Kramer N.F., Giese H., Sørensen J.L. (2016). Fast Screening of Antibacterial Compounds from Fusaria. Toxins.

[22] Jarolim K., Wolters K., Woelflingseder L., Pahlke G., Beisl J., Puntscher H., Braun D., Sulyok M., Warth B., Marko D. (2018). The secondary Fusarium metabolite aurofusarin induces oxidative stress, cytotoxicity and genotoxicity in human colon cells. Toxicol. Lett..

[23] Cox J., Mann M. (2011). Quantitative, High-Resolution Proteomics for Data-Driven Systems Biology. Annu. Rev. Biochem..

[24] Lazar C., Gatto L., Ferro M., Bruley C., Burger T. (2016). Accounting for the Multiple Natures of Missing Values in Label-Free Quantitative Proteomics Data Sets to Compare Imputation Strategies. J. Proteome Res..

[25] Smith S.M. (2011). Strategies for the purification of membrane proteins. Methods Mol. Biol..

[26] Schwanhüusser B., Busse D., Li N., Dittmar G., Schuchhardt J., Wolf J., Chen W., Selbach M. (2011). Global quantification of mammalian gene expression control. Nature.

[27] Zhou F., Lu Y., Ficarro S.B., Adelmant G., Jiang W., Luckey C.J., Marto J.A. (2013). Genome-scale proteome quantification by DEEP SEQ mass spectrometry. Nat. Commun..

[28] Finn R.D., Attwood T.K., Babbitt P.C., Bateman A., Bork P., Bridge A.J., Chang H.-Y., Dosztanyi Z., El-Gebali S., Fraser M. (2017). InterPro in 2017-beyond protein family and domain annotations. Nucleic Acids Res..

[29] Finn R.D., Coggill P., Eberhardt R.Y., Eddy S.R., Mistry J., Mitchell A.L., Potter S.C., Punta M., Qureshi M., Sangrador-Vegas A. (2016). The Pfam protein families database: Towards a more sustainable future. Nucleic Acids Res..

[30] Josefsen L., Droce A., Sondergaard T.E., Sørensen J.L., Bormann J., Schäfer W., Giese H., Olsson S. (2012). Autophagy provides nutrients for nonassimilating fungal structures and is necessary for plant colonization but not for infection in the necrotrophic plant pathogen *Fusarium graminearum*. Autophagy.

[31] Hansen F.T., Droce A., Sørensen J.L., Fojan P., Giese H., Sondergaard T.E. (2012). Overexpression of NRPS4 leads to increased surface hydrophobicity in *Fusarium graminearum*. Fungal Biol..

[32] Berger S.T., Ahmed S., Muntel J., Cuevas Polo N., Bachur R., Kentsis A., Steen J., Steen H. (2015). MStern Blotting-High Throughput Polyvinylidene Fluoride (PVDF) Membrane-Based Proteomic Sample Preparation for 96-Well Plates. Mol. Cell. Proteomics.

[33] Herbst F.-A., Danielsen H.N., Wimmer R., Nielsen P.H., Dueholm M.S. (2015). Label-free quantification reveals major proteomic changes in Pseudomonas putida F1 during the exponential growth phase. Proteomics.

[34] Tyanova S., Temu T., Cox J. (2016). The MaxQuant computational platform for mass spectrometry-based shotgun proteomics. Nat. Protoc..

[35] Cox J., Hein M.Y., Luber C.A., Paron I., Nagaraj N., Mann M. (2014). Accurate proteome-wide label-free quantification by delayed normalization and maximal peptide ratio extraction, termed MaxLFQ. Mol. Cell. Proteom..

[36] (2017). UniProt: The universal protein knowledgebase. Nucleic Acids Res..

[37] Tyanova S., Temu T., Sinitcyn P., Carlson A., Hein M.Y., Geiger T., Mann M., Cox J. (2016). The Perseus computational platform for comprehensive analysis of (prote)omics data. Nat. Methods.

[38] Wider G., Dreier L. (2006). Measuring protein concentrations by NMR spectroscopy. J. Am. Chem. Soc..

[39] Cappellini R.A., Peterson J.L. (1965). Macroconidium Formation in Submerged Cultures by a Non-Sporulating Strain of Gibberella zeae. Mycologia.

[40] Leslie J.F., Summerell B.A., Leslie J.F., Summerell B.A. (2007). The Fusarium Laboratory Manual.

[41] Sørensen J.L., Sondergaard T.E. (2014). The effects of different yeast extracts on secondary metabolite production in Fusarium. Int. J. Food Microbiol..

[42] Sorensen J.L., Giese H. (2013). Influence of carbohydrates on secondary metabolism in *Fusarium avenaceum*. Toxins.

