# Supplementary material for: Enhancing the Production of the Fungal Pigment Aurofusarin in Fusarium graminearum
Source: Toxins (Basel). 2018 Nov 21;10(11):485. doi: 10.3390/toxins10110485 (PMC6266765; doi:10.3390/toxins10110485)
Supplement: Supplementary file 1 [file toxins-10-00485-s001.pdf]

Toxins **2018**, *10*, 485; doi:10.3390/toxins10110485

Supplementary Materials: Enhancing the Production of the Fungal Pigment Aurofusarin in *Fusarium graminearum*

Klaus Ringsborg Westphal, Rasmus Dam Wollenberg, Florian-Alexander Herbst, Jens Laurids Sørensen, Teis Esben Sondergaard and Reinhard Wimmer

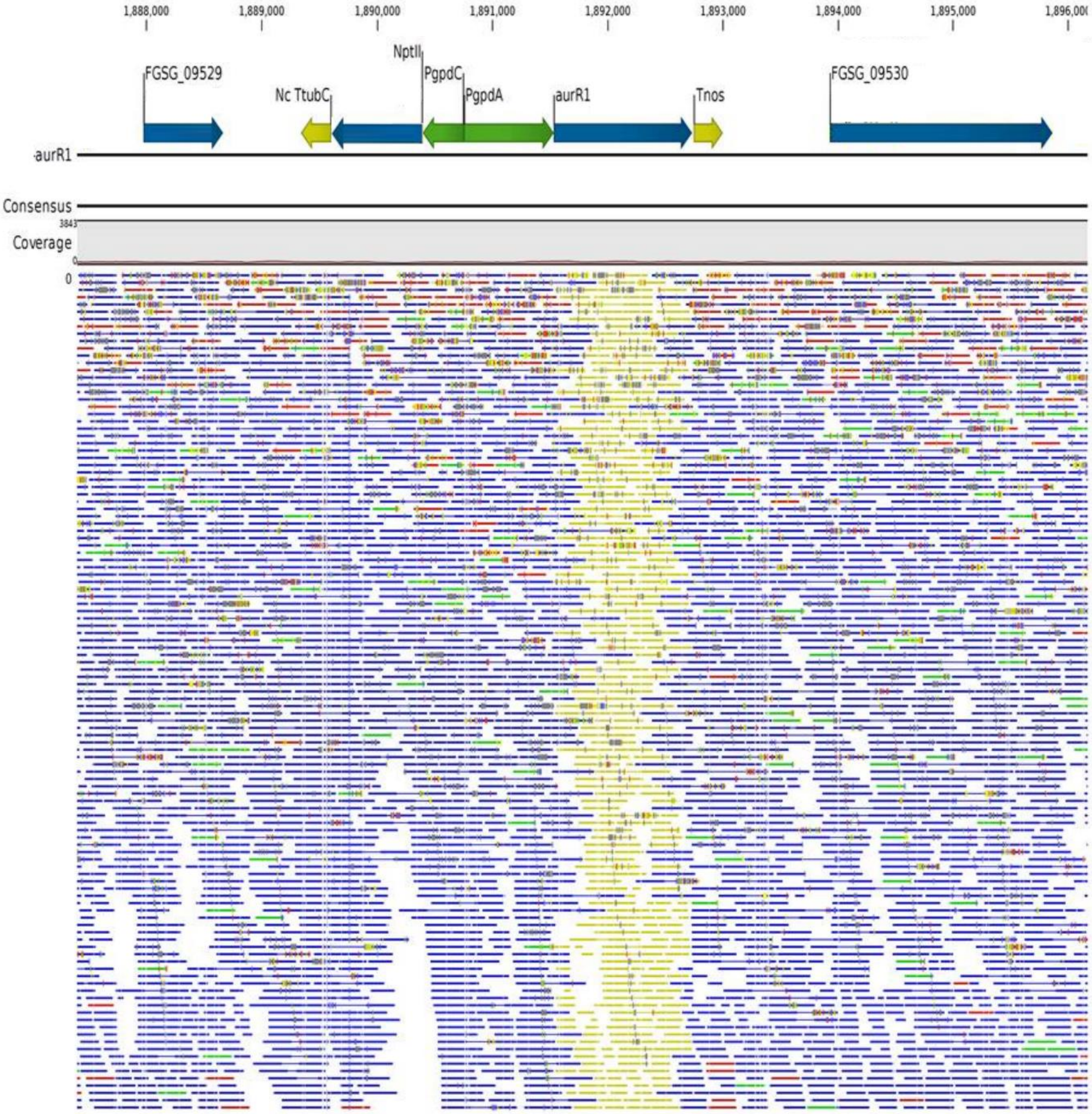

Figure S1. Sequencing results for the OE::*aurR1* mutant. (**Top**) *In silico* annotations at the tubulin locus of *Fusarium graminearum* with inserted resistance gene (*nptII*) and *aurR1*. Green, yellow, and blue annotations indicate promotors, terminators, and genes, respectively. (**Bottom**) Read-mappings from full genome sequencing represented as either paired-end (blue), sense strand (green), anti-sense strand (red), or mapped to other places in the genome (yellow).

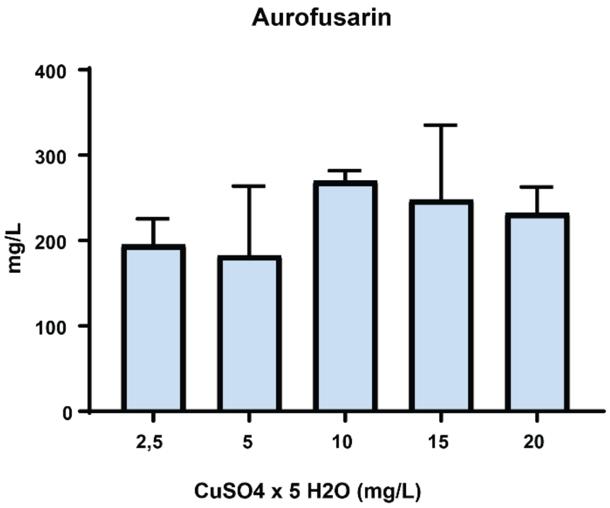

Figure S2. Production of aurofusarin in the OE::*aurR1* mutant in response to variable concentrations of $CuSO_4$ x 5 H_2O in the growth medium. The bars represent mean values from four replicates with error bars showing the standard deviation.
